# Dependence of Positive Bias Stress Instability on Threshold Voltage and Its Origin in Solution-Processed Aluminum-Doped Indium Oxide Thin-Film Transistors

**DOI:** 10.3390/nano14050466

**Published:** 2024-03-04

**Authors:** Jeong-Hyeon Na, Jun-Hyeong Park, Won Park, Junhao Feng, Jun-Su Eun, Jinuk Lee, Sin-Hyung Lee, Jaewon Jang, In Man Kang, Do-Kyung Kim, Jin-Hyuk Bae

**Affiliations:** School of Electronic and Electrical Engineering, Kyungpook National University, 80 Daehakro, Bukgu, Daegu 41566, Republic of Korea; jh2jh3@naver.com (J.-H.N.); jeef12345@naver.com (J.-H.P.); qkrdnjs0320@naver.com (W.P.); junhaosuhua@gmail.com (J.F.); wnstn0812@naver.com (J.-S.E.); kein98@knu.ac.kr (J.L.); sinhlee@knu.ac.kr (S.-H.L.); j1jang@knu.ac.kr (J.J.); imkang@ee.knu.ac.kr (I.M.K.)

**Keywords:** oxide semiconductor, thin-film transistors (TFTs), bias instability, threshold voltage dependency, degradation mechanism

## Abstract

The initial electrical characteristics and bias stabilities of thin-film transistors (TFTs) are vital factors regarding the practical use of electronic devices. In this study, the dependence of positive bias stress (PBS) instability on an initial threshold voltage (V_TH_) and its origin were analyzed by understanding the roles of slow and fast traps in solution-processed oxide TFTs. To control the initial V_TH_ of oxide TFTs, the indium oxide (InO_x_) semiconductor was doped with aluminum (Al), which functioned as a carrier suppressor. The concentration of oxygen vacancies decreased as the Al doping concentration increased, causing a positive V_TH_ shift in the InO_x_ TFTs. The V_TH_ shift (∆V_TH_) caused by PBS increased exponentially when V_TH_ was increased, and a distinct tendency was observed as the gate bias stress increased due to a high vertical electric field in the oxide dielectric. In addition, the recovery behavior was analyzed to reveal the influence of fast and slow traps on ∆V_TH_ by PBS. Results revealed that the effect of the slow trap increased as the V_TH_ moved in the positive direction; this occured because the main electron trap location moved away from the interface as the Fermi level approached the conduction band minimum. Understanding the correlation between V_TH_ and PBS instability can contribute to optimizing the fabrication of oxide TFT-based circuits for electronic applications.

## 1. Introduction

Oxide thin-film transistors (TFTs) have been considered to be the building blocks for various next-generation electronic devices owing to their advantages, such as low processing temperature, high mobility, device-to-device uniformity, optical transparency, and high mechanical flexibility [1,2,3]. Large-area organic light-emitting diode (OLED) displays with indium–gallium–zinc-oxide TFTs have been commercialized [4,5]. Recently, the use of oxide TFTs in displays and monolithic three-dimensional integration has been actively studied [6]. The commercial feasibility of TFTs depends on their initial electrical characteristics. Among the various electrical parameters, such as mobility, subthreshold swing (SS), on/off current ratio (I_on/off_), and threshold voltage (V_TH_), V_TH_ is vital because it determines the depletion or enhancement mode of TFTs. Depletion- and enhancement-mode TFTs should be appropriately selected based on the product’s characteristics [7,8]. In addition, V_TH_ should be effectively controlled to greater or less than 0. For example, when configuring a compensation circuit within a pixel in a display, the compensation error may increase if V_TH_ is too high or low [9,10]. In addition, a positive V_TH_ is required to reduce circuit design complexity, as well as power consumption, when it is used as a gate driver circuit [11,12]. Thus, V_TH_ is an important initial parameter and should be effectively controlled during fabrication.

Based on their commercial usage, the robustness of TFT devices and their temperature, light, humidity, and bias stabilities must be ensured. Various external stresses, such as light or humidity, can be partially blocked by appropriately designing the device’s structure, as well as introducing additional layers [13,14]. However, blocking the bias stress in TFTs is difficult as the electrical bias applied to drive the transistors is an internal stress. Therefore, the origin of bias stability must be studied to determine the robustness of the device. Extreme V_TH_ shifts and SS degradation occurs when negative gate bias and illumination stress are simultaneously applied [15,16,17]. When the oxide TFTs act as a switch rather than the current supplier to the OLEDs in displays, negative bias illumination stress (NBIS) is critical because the switching TFTs are typically turned off by negative gate bias [18]. However, the NBIS can be improved through the reduction of oxygen vacancies or peroxides, or introducing light-blocking metals [19]. Recently, oxide TFTs have been used in OLED displays [20], and positive bias stress (PBS) is becoming increasingly important because it is applied to the driving transistor that supplies a current to the OLED and gate driver circuit [18]. Although initial V_TH_ and PBS must be simultaneously considered, their correlation is not entirely clear. The PBS stability can be further improved based upon the initial V_TH_. Moreover, solution-processed oxide TFTs have been actively studied owing to their advantages of high scalability and throughput [21,22,23]. However, they have poorer electrical stabilities than those fabricated by conventional sputtering because of the high density of physical and chemical defects that are generated during fabrication [24,25]. Therefore, the relation between V_TH_ and PBS stability must be further deduced. Additionally, VTH must be decreased in solution-processed oxide TFTs with relatively poor PBS stability than the sputtered oxide TFTs.

In this study, the correlation between an initial V_TH_ and PBS instability in solution-processed oxide TFTs was investigated. The degradation mechanism of PBS based on V_TH_ was demonstrated through the observation of the recovery behavior of oxide TFTs after PBS. Various molarities of Al were doped in the InO_x_ semiconductor to examine the dependency of V_TH_ on PBS instability; V_TH_ was successfully controlled in a wide range. The V_TH_ shift of Al-doped oxide TFTs was determined via the chemical and optical analysis of InO_x_ and indium-aluminum-oxide (InAlO_x_) thin-film characteristics. Through measuring the PBS and the recovery of oxide TFTs, it was found that the initial V_TH_ and ∆V_TH_ induced by PBS have an exponential relation, regardless of the gate bias stress (V_G,stress_). Moreover, analysis of the recovery behavior revealed that the role of slow traps in ΔV_TH_ became dominant in oxide TFTs with positive V_TH_.

## 2. Materials and Methods

### 2.1. Preparation of Oxide Precursor Solutions

InO_x_ and InAlO_x_ precursor solutions were prepared for fabricating oxide semiconductor thin films. A 0.1 M InO_x_ precursor solution was prepared by dissolving In(NO_3_)_3_·*x*H_2_O (Sigma-Aldrich, St. Louis, MO, USA) in 2-methoxyethanol (2-ME), which is generally used as a solvent for oxide precursor solutions [26,27]. To control the initial V_TH_, InAlO_x_ precursor solutions were prepared by adding 0.005, 0.010, and 0.015 M Al(NO_3_)_3_·9H_2_O (Sigma-Aldrich, St. Louis, MO, USA) to 0.1 M InO_x_ precursor solution. The molar ratio of Al/In of the three prepared solutions are 0.05, 0.10, and 0.15. All solutions were stirred at 50 °C for 12 h.

### 2.2. Fabrication of Devices

**Figure 1:**
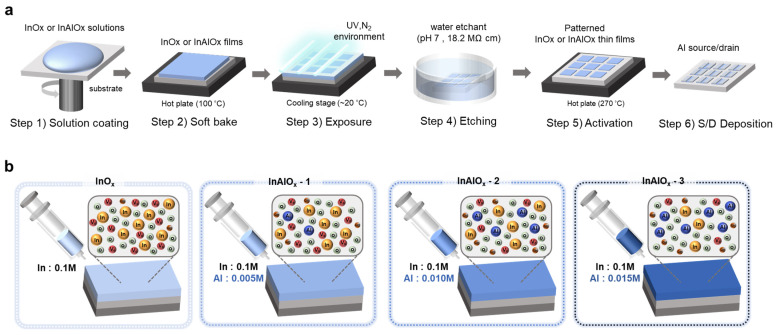
(**a**) Schematic of the device fabrication process. (**b**) Schematic of the InAlO_x_ thin films with various Al doping concentration.

Si/SiO_2_ wafer substrates were prepared for gates and gate dielectrics. Wafers were cleaned via sonication for 10 min each in acetone, isopropyl alcohol, and deionized water. Then, the substrates were dried in an N_2_ atmosphere, and annealed at 300 °C for 5 min to remove any residual moisture. The water etchant–based photo patterning method was used to deposit the patterned oxide semiconductor for the fabrication of high-performance oxide TFTs [28]. Figure 1a schematizes the fabrication process of InO_x_ and InAlO_x_ thin films. The precursor solutions were spin-coated at 3000 rpm for 20 s onto a cleaned Si/SiO_2_ wafer (Step 1). The precursor solution-deposited wafer was soft baked at 100 °C for 30 s (Step 2). Then, a fine metal mask was placed on the wafer to irradiate ultraviolet (UV) light in a selective area. The UV was exposed to InO_x_ for 120s and InAlO_x_ for 150 s under an N_2_ atmosphere (Step 3). UV irradiation (25 mW cm^−2^) was conducted via a low-pressure mercury lamp with two main wavelengths, namely 253.7 (90%) and 184.9 nm (10%). UV photons induced the decomposition of nitrate ligand via photochemical cleavage. Then, the wafer was etched in a deionized water etchant for 1 min (Step 4). Patterned InO_x_ and InAlO_x_ were annealed at 100 °C for 10 min and 270 °C for 2 h, respectively, on a hot plate in the air (Step 5). In this series of processes, hydrolysis and condensation reactions were promoted, which yielded low-defect high-quality oxide thin films. Lastly, a 50-nm-thick Al film was deposited for source and drain electrodes using thermal evaporation (Step 6). The channel length (L) and width (W) were 100 and 1000 μm, respectively. The patterned thin films or TFTs fabricated using InO_x_, InAlO_x_ with 0.005 M Al doping, InAlO_x_ with 0.010 M Al doping, and InAlO_x_ with 0.015 M Al doping were named InO_x_, InAlO_x_-1, InAlO_x_-2, and InAlO_x_-3, respectively, as shown in Figure 1b.

### 2.3. Analysis of Thin Films and Devices

Chemical compositions of InO_x_ and InAlO_x_ thin films were investigated using X-ray photoelectron spectroscopy (XPS; ThermoFisher Scientific, NEXSA, Waltham, MA, USA) with an Al Kα (1486.6 eV) light source. To determine the optical properties, such as the bandgap, of the deposited oxide semiconductor films, a UV–visible (UV–Vis) spectrophotometer (Perkin Elmer, Waltham, MA, USA, LAMBDA 265) was used. The InO_x_ and InAlO_x_ films deposited on cleaned glass were measured by a transmittance mode. A probe station (MS Tech, Oak Ridge, TN, USA, MST-6VC) was used to measure the transfer curves of InO_x_ TFTs under temperature variations. The electrical characteristics of the devices were obtained using a semiconductor parameter analyzer (4200-SCS, Keithley, Cleveland, OH, USA) combined with an ultrafast I–V module (4225-PMU, Keithley). All devices were measured at room temperature in a dark environment.

## 3. Results

**Figure 2:**
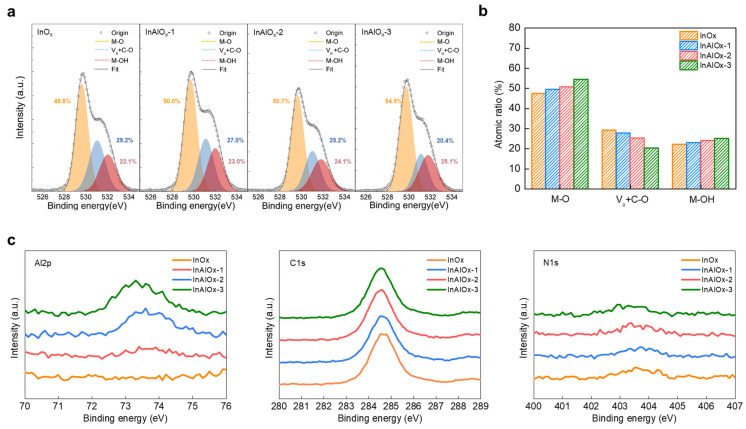
(**a**) XPS O 1s spectra for InO_x_ and InAlO_x_ semiconductors with various Al doping concentrations. (**b**) The comparison of the atomic percentages of M–O, Vo + C–O, and M–OH of InAlO_x_ semiconductors with various Al doping concentrations. (**c**) XPS Al 2p, C 1s, and N 1s spectra for InO_x_ and InAlO_x_ semiconductors with various Al doping concentrations.

InO_x_ and InAlO_x_ thin films with various molarities were fabricated to demonstrate their electrical characteristics, as shown in Figure 1a. The changes in the atomic structure with increasing Al doping concentration were evaluated via XPS analysis. Figure 2a shows the O1s spectra of InO_x_, InAlO_x_-1, InAlO_x_-2, and InAlO_x_-3. The oxygen bonding states were analyzed via deconvolution into three Gaussian Lorentzian peaks corresponding to the oxygen species of the metal–oxide (M–O), oxygen vacancy (V_o_), and metal–hydroxide (M-OH) centered at ~529.6, ~531.0, and ~531.8 eV, respectively [29,30,31]. The oxygen-binding energy of Al was higher than that of In; therefore, Al suppressed the oxygen defects in the active layer [32]. As shown in Figure 2b, as the Al doping concentration increases, M–O bonding increases and V_o_ decreases, thereby increasing the carrier concentration [33]. The M–OH bonding increases with the Al doping concentration, which is explained via the M–OH condensation reaction. At a certain temperature, two M–OH bonds form an M–O–M bond via condensation; however, the reaction does not progress when Al is doped. Figure 2c shows the binding energy of the element extracted from XPS. As the Al doping concentration increases, the Al2p peak can be clearly observed. In step 2 (Figure 1a), a large amount of nitrate ligands in the oxide film disappears; in step 3 (Figure 1a), the nitrate ligands inside the film are decomposed by UV irradiation, hydroxyl radical species, and M−OH bond formation [28]. Therefore, the N1s peak was not clear for all the TFT devices. The C–O/C–OH bond with a binding energy of ~287.0 eV was decomposed via UV irradiation, and only the C–H/C–C bond with a binding energy of ~285.0 eV remained; all TFT devices showed the same c1s peak [34]. 

**Figure 3:**
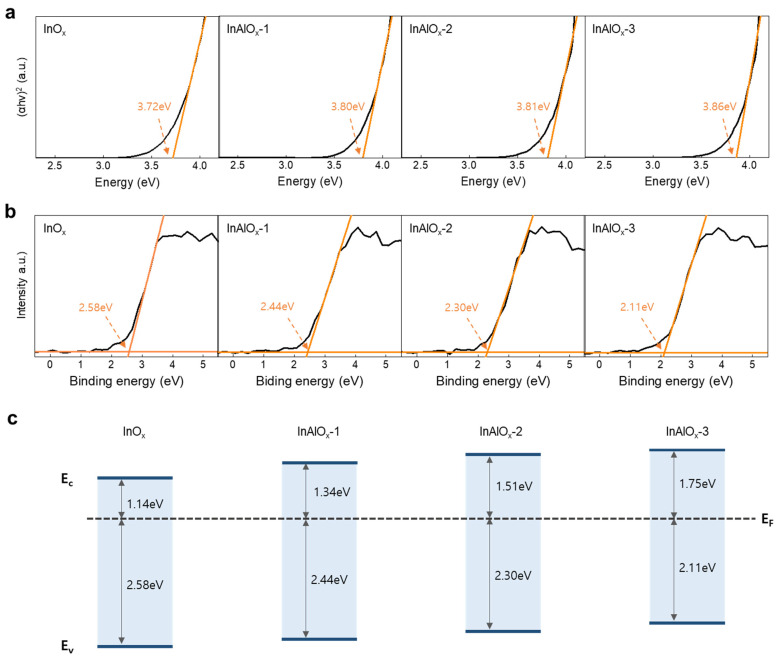
(**a**) Optical bandgap and (**b**) valence band offset spectra for InO_x_ and InAlO_x_ semiconductors with various Al doping concentrations. (**c**) Energy band alignment of InO_x_ and InAlO_x_ semiconductors with various Al doping concentrations.

To understand the charge transport mechanism, the interfacial energy-level alignments between the active layer and source/drain electrode must be elucidated. The energy band alignment of InO_x_ and InAlO_x_ thin films was investigated via UV–Vis spectrophotometry and XPS analysis. The optical bandgap (E_g_) was obtained from the extrapolated linear fit of (αh*ν*)^2^ versus the photon energy, as shown in Figure 3a. The extracted E_g_ values of InO_x_, InAlO_x_-1, InAlO_x_-2, and InAlO_x_-3 were 3.72, 3.80, 3.81, and 3.86 eV, respectively. The atomic radius of In was 156 pm, larger than that of Al. Thus, the substitution of a smaller atom (Al) in the composition with a larger atom (In) decreases the E_g_; these results coincide with the theoretical expectations [35]. As shown in Figure 3b, as the Al doping concentration increases, the valence band offset decreases from 2.58 to 2.11. Thus, with increasing Al doping concentration, E_g_ increases and valence band offset decreases, thereby increasing the conduction band offset (Figure 3c). This result suggests that as the Al doping concentration increases, V_o_ and carrier concentration decrease [36,37]. 

**Figure 4:**
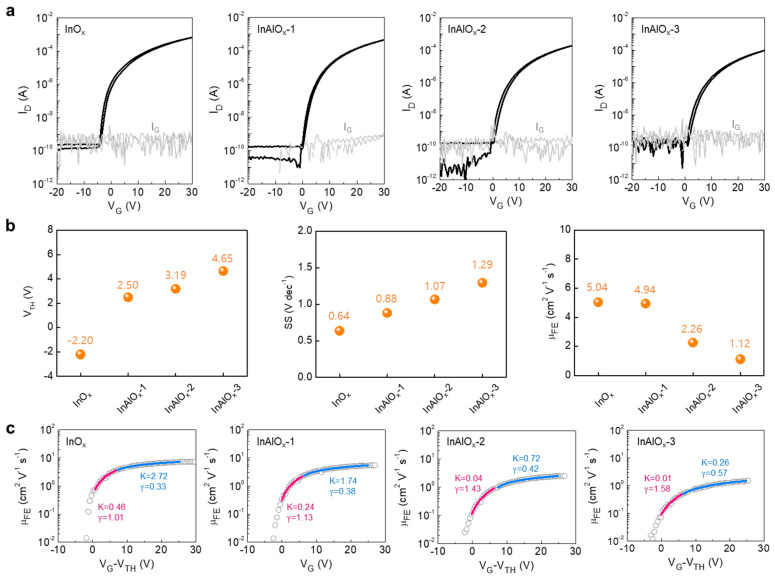
(**a**) Transfer characteristics of InAlO_x_ TFTs with various Al doping concentrations. (**b**) Electrical parameters including threshold voltage, subthreshold swing, and field-effect mobility of InAlO_x_ TFTs with various Al doping concentrations. (**c**) Field-effect mobility versus overdrive voltage of InAlO_x_ TFTs with various Al doping concentrations.

To demonstrate the electrical characteristics based on the carrier concentration of the oxide semiconductor, TFTs containing InO_x_, InAlO_x_-1, InAlO_x_-2, and InAlO_x_-3 with a bottom-gate, top-contact structure were fabricated. Figure 4a shows the transfer characteristics of InO_x_, InAlO_x_-1, InAlO_x_-2, and InAlO_x_-3. Gate voltage (V_G_) swept from −20 to 30 V with a 0.5 V step, and drain voltage (V_D_) was fixed at 30 V. The gray line shows the gate leakage current (I_G_). As shown in the transfer curves, all TFTs had acceptable switching characteristics with low off current levels of 10^−12^–10^−11^ A, regardless of the doping concentration. In contrast, the curves positively shifted, and on current gradually decreased as the Al doping concentration increased, which originated from the decrease in the carrier concentration of semiconductors as the Al doping concentration increased. For a more detailed comparison, the summarized results of the electrical parameters of InO_x_ and InAlO_x_ TFTs are shown in Figure 4b. Here, V_TH_ was defined as the value of V_G_ that induces drain current (I_D_) = 10 nA × W/L at V_D_ = 30 V. A positive shift of V_TH_, increase in SS, and decrease in field-effect mobility in saturation region (μ_FE_) was observed. A positive shift of V_TH_ and a decrease in μ_FE_ are reasonable because the Al doping suppresses the formation of V_o_, thereby decreasing the carrier concentration, as confirmed by the XPS results. In addition, the maximum trap density (*N_T_*_,max_) increased with Al doping concentration due to an increase in the SS based on the following equation:

(1) $$SS=\frac{{qk}_{B\;}T(N_{T}t_{ch}+D_{it})}{C_{ox}\log\left(e\right)},$$

where *k*, *q*, *T*, and C_i_ are the Boltzmann constant, elementary electron charge, and absolute temperature, respectively. *N_T_* and *D_it_* are the number of fast bulk traps and semiconductor-insulator interfacial traps. *N_T_*_,max_ of InO_x_, InAlO_x_-1, InAlO_x_-2, and InAlO_x_-3 are 4.66, 6.11, 6.96, 7.96 × 10^18^ cm^−3^ eV^−1^, respectively.

Unlike single-crystal Si-based transistors that follow the band transport theory, oxide semiconductor–based transistors have a different charge transport mechanism due to their atomic structural disorder. In general, the current–voltage behavior of oxide TFTs is modeled based on trap-limited conduction (TLC) and percolation [38,39]. In particular, the charge transport mechanism varies based on the electron concentration in the semiconductor, which is identified through the power-law dependence of mobility [40]. Here, a power-law equation was employed in the (*V_G_*-V_TH_)—*μ*_FE_ curve to investigate the influence of Al doping on carrier trapping and thermal release at the tail states and percolation in the above-threshold regime in oxide TFTs, as shown in Figure 4c.

(2) $$\mu_{FE}={K(V_{G}-V_{T,P})}^{\gamma },$$

where *V*_P_ is the percolation voltage, and *K* and γ are related to the nature of the electron transport. In a typical power-law model, TLC affects the constant *K*, and percolation conduction determines the exponent γ [41]. To observe the transition of the electron conduction mechanism by *V*_G_, each curve was fitted in the low *V*_G_ and high *V*_G_ regions by the power-law equation. In low *V*_G_ region, the *K* values of InO_x_, InAlO_x_-1, InAlO_x_-2, and InAlO_x_-3 are 0.46, 0.24, 0.04, and 0.01, respectively. This suggests that the TLC prevails as the initial V_TH_ positively shifts in the oxide TFTs. As V_TH_ shifts positively in the low *V*_G_ region, γ also tends to increase, suggesting that electron conduction is greatly restricted by the potential barrier. Meanwhile, in high *V*_G_ region, *K* and γ values according to Initial V_TH_ show the same tendency as those in the low V_G_ region. Interestingly, *K* and γ overall show higher and lower values, respectively, compared to the low *V*_G_ region. This implies that charge transport by percolation is dominant rather than the tail states as the *V*_G_ increase. 

**Figure 5:**
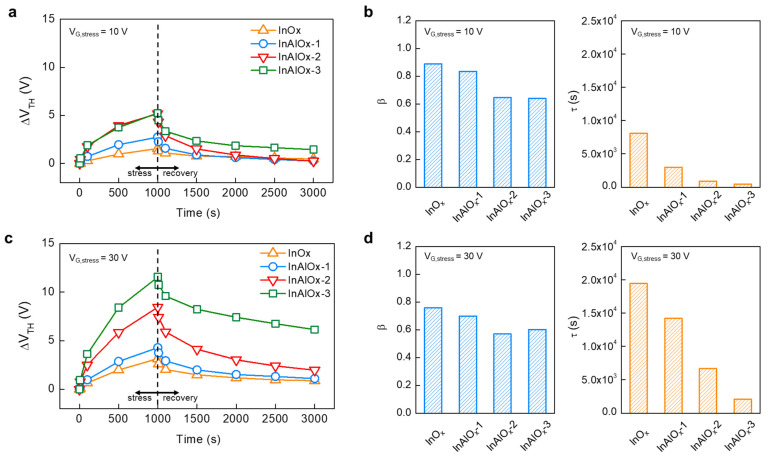
(**a**) Time evolution of ΔV_TH_ during the low V_G_ stress (V_G_ = 10 V, V_D_ = 0 V, t = 1000 s) and subsequent recovery (V_G_ = 0 V, V_D_ = 0 V, t = 2000 s) phases, and (**b**) corresponding stretched exponential function parameters of InAlOx TFTs with various Al doping concentrations. (**c**) Time evolution of ΔV_TH_ during the high V_G_ stress (V_GS_ = 30 V, V_DS_ = 0 V, t = 1000 s) and subsequent recovery (V_GS_ = 0 V, V_DS_ = 0 V, t = 2000 s) phases and (**d**) corresponding stretched-exponential function parameters of InAlO_x_ TFTs with various Al doping concentrations.

The PBS stability, along with the initial characteristics of the device, should be considered for the practical use of the device. Then, to verify the influence of Al doping concentration on the PBS-induced instability, V_TH_ characteristics were determined based on the PBS and recovery. Figure 5 shows the time evolution of V_TH_ shift (∆V_TH_) during PBS and subsequent recovery. For PBS, a V_G,stress_ of 10 or 30 V was applied for 1000 s. To avoid TFT degradation and asymmetric charge trapping due to current stress, a V_D_ of 0 V was applied during stress. Immediately after 1000 s of PBS, recovery was followed for 2000 s at V_G_ = 0 V and V_D_ = 0 V. As shown in Figure 5a, V_TH_ shifted positively during PBS with a V_G,stress_ of 10 V, and the degree of recovery tended to increase with Al doping concentration. V_TH_ recovers rapidly in the early stages of recovery after stress and then tends to saturate [42,43]. InO_x_ TFTs recovered at the fastest rates, and after recovery for 2000 s, V_TH_ returned close to its initial state. InAlO_x_-3 with a high Al doping concentration showed the slowest recovery speed and a high ∆V_TH_, even after recovery at 2000 s. This characteristic means that some of the electrons trapped in the interface and gate dielectric by PBS are easily detrapped by the recovery phase, but the rest remain without being detrapped. Moreover, the results show that these trap/detrap characteristics differ depending on the Al doping concentration.

The stretched-exponential time dependence of ∆V_TH_ suggests that ∆V_TH_ originated from electron trapping at the trap sites [43,44]. The stretched-exponential equation for ∆V_TH_(t) is as follows:

(3) $$∆V_{TH}\left(t\right)=∆V_{TH0}\{1-exp[-\left({t/\tau )}^{\beta }\right]\},$$

where ∆V_TH0_ is the ∆V_TH_ at infinite time, *β* is the stretched-exponential exponent, and *τ* is the characteristic trapping time of carriers. ∆V_TH_ by PBS with a V_G,stress_ of 10 V for InO_x_ and InAlO_x_ TFTs were fitted to the stretched-exponential equation, and *β* and *τ* for each device were extracted, as shown in Figure 5b. *τ* decreased as the Al doping concentration increased, implying that electrons were easily trapped in the trap site. Figure 5c,d show the time evolution of ∆V_TH_ by PBS with a V_G,stress_ of 30 V and subsequent recovery, and the corresponding *β* and *τ* during PBS, respectively. ∆V_TH_ at a V_G,stress_ of 30 V shows a similar trend to the PBS with V_G,stress_ = 10 V, as shown in Figure 5a,b. Notably, as V_G,stress_ increases, ΔV_TH_ becomes larger in the same device and the correlation between Al doping concentration and ΔV_TH_ becomes clearer. Through a quantitative comparison of *τ*, it is confirmed that charge trapping occurs more effectively with strong vertical electric field as V_G,stress_ increases.

**Figure 6:**
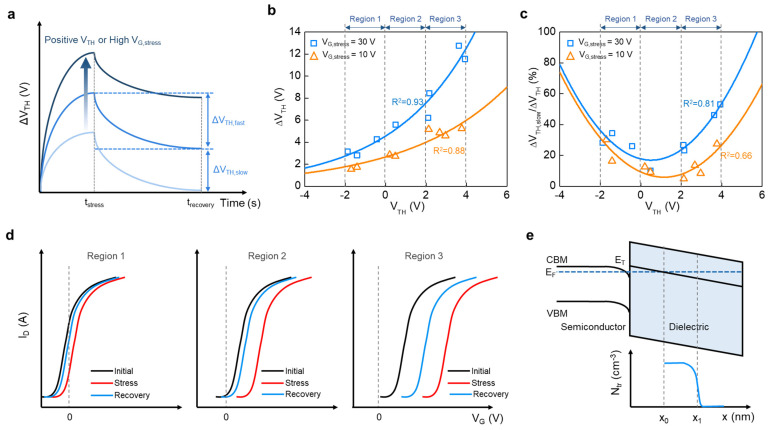
Dependence of (**a**) time evolution of ΔV_TH_ and corresponding slow trapping (ΔV_TH,slow_) and fast trapping (ΔV_TH,fast_) components. (**b**) ΔV_TH_ and (**c**) ΔV_TH,slow_/ΔV_TH_ on the initial V_TH_ of oxide TFTs. Two devices each from InO_x_, InAlO_x_-1, InAlO_x_-2, and InAlO_x_-3 were measured. (**d**) Schematic of transfer characteristics at initial state, after stress, and after recovery of oxide TFTs in regions 1, 2, and 3. (**e**) The energy band of oxide TFTs and extracted trapped electron distribution in the fast and deep gate dielectric traps.

Based on the initial electrical characteristics according to the Al doping concentration in InO_x_ TFTs, the influence of initial V_TH_ on ∆V_TH_ is further revealed. Figure 6a depicts the time evolution of ∆V_TH_. Al-doped InO_x_ TFTs show higher ∆V_TH_ as V_TH_ shifts more positively, or as V_G,stress_ increases (Figure 5). Here, electrons that are not easily detrapped during the recovery phase are considered as slow traps, whereas those that are detrapped during the recovery phase are treated as fast traps [42]. Variations in ∆V_TH_ based on V_TH_ were plotted by increasing the number of measured samples to clearly identify the correlation between V_TH_ and ∆V_TH_, as shown in Figure 6b. Regardless of V_G,stress_, ∆V_TH_ tended to increase exponentially as V_TH_ increased. The ∆V_TH_ by PBS with 10 and 30 V for devices with a V_TH_ in the range of −2–4 V was fitted with an exponential function, and showed high R^2^ values of 0.88 and 0.93, respectively. As V_G,stress_ increased from 10 to 30 V, the trend became clear. ∆V_TH,slow_ was extracted from the recovery behavior, and the ratio of slow trap according to V_TH_ is shown in Figure 6c. In enhancement-mode TFTs with a V_TH_ of 0 V or higher, ∆V_TH,slow_/∆V_TH_ tended to increase exponentially as V_TH_ increased, whereas in depletion-mode TFTs, an opposite trend was confirmed. This trend could be fitted with a parabola function and showed high R^2^ values of 0.66 and 0.81 when V_G,stress_ was 10 and 30 V, respectively. PBS with a V_G,stress_ of 30 V had a higher slow trap rate than when that with a V_G,stress_ of 10 V.

To estimate the charge transport mechanism, we divided the plot of ∆V_TH_ against V_TH_ into three regions, with V_TH_ of −2–0 V, 0–2 V, and 2–4 V named as regions 1, 2, and 3, respectively (Figure 6a,b). Figure 6d illustrates the transfer characteristics at the initial state, after stress, and after recovery by region. From Region 1 to 3, the initial V_TH_ shifts positively and shows a large ∆V_TH_ after stress. This ∆V_TH_ dependence on V_TH_ can be explained based on the band diagram, where x_0_ is the intersection of the Fermi level (E_F_) and trap level (E_T_), and most electrons are trapped between x_0_ and x_1_, as shown in Figure 6e [45,46]. As shown in Figure 3c, as the Al doping concentration increases, E_F_ approaches the conduction band minimum. Thus, x_1_, where the injected charge is mainly located, moves away from the interface based on the function of the electron transfer distance [45]. In other words, electrons trapped deep cannot be easily detrapped through recovery at room temperature, and as V_TH_ shifts positively, the rate of slow trap increases. Meanwhile, ∆V_TH_ increases with V_G_ and stress caused by x_1_ moving away from the semiconductor–dielectric interface as the electric field applied to the oxide dielectric increases, implying that the electrons are trapped deeper [46]. Notably, compared to ∆V_TH_, the degree of recovery in region 1 was less than that in region 2. This is because depletion-mode TFTs do not form a flat band at V_G_ = 0 V, but still form charge accumulation by band bending, forming x_0_. In addition, region 2 showed smaller ∆V_TH_ and higher recovery characteristics compared to region 3. These results suggest that as the initial V_TH_ increases or the carrier concentration of the semiconductor decreases, the influence of the slow trap on PBS degradation increases. Thus, an inappropriate positive V_TH_ may considerably deteriorate the short- and long-term stabilities.

## 4. Conclusions

The linearity and resolution of the delay line has a great effect on transmitter performance. In order to overcome the bottleneck of low linearity and low resolution, an improved delay line structure is proposed with a calibration algorithm to conquer PVT variations for this all-digital design. Measurement results show that the proposed structure with the calibration algorithm can evidently improve the linearity and resolution of the delay line. In summary, we demonstrated the correlation between V_TH_ and ∆V_TH_ in solution-processed oxide TFTs. The oxygen vacancy and bandgap were controlled by tuning the Al doping concentration in the Al-doped InO_x_, which changed the initial V_TH_ of the oxide TFTs. As V_TH_ increased, ∆V_TH_ due to PBS increased exponentially, and the deterioration became more severe as the V_G,stress_ increased from 10 to 30 V. Using the stretched-exponential function, it was revealed that the positive V_TH_ and high V_G,stress_ decreased the characteristic trapping time of carriers, implying that the charge trapping occurs effectively. Based on the recovery characteristics after PBS, the slow trap acts dominantly to ∆V_TH_, and trapped electrons are not easily detrapped under a high V_G,stress_ or in TFTs with a positive V_TH_. In case of PBS with a V_G,stress_ of 30 V, ∆V_TH,slow_/∆V_TH_ showed a significant difference of 10.07% and 53.12% for TFTs with V_TH_ of 0.49 and 3.93 V, respectively. The results of this study can be used to determine the initial V_TH_ considering PBS and improving PBS in solution-processed oxide TFTs.

## Data Availability

Data are contained within the article.
